# Principal component analysis of flow-volume curves in COPDGene to link spirometry with phenotypes of COPD

**DOI:** 10.1186/s12931-023-02318-4

**Published:** 2023-01-19

**Authors:** Kenneth Verstraete, Nilakash Das, Iwein Gyselinck, Marko Topalovic, Thierry Troosters, James D. Crapo, Edwin K. Silverman, Barry J. Make, Elizabeth A. Regan, Robert Jensen, Maarten De Vos, Wim Janssens

**Affiliations:** 1 Laboratory of Respiratory Diseases and Thoracic Surgery (BREATHE), Department of Chronic Diseases and Metabolism, KU Leuven, Herestraat 49, O&N 1Bis, Box 706, 3000 Leuven, Belgium; 2 STADIUS Center for Dynamical Systems, Signal Processing and Data Analytics, Department of Electrical Engineering (ESAT), KU Leuven, Leuven, Belgium; 3 ArtiQ NV, Leuven, Belgium; 4 Department of Rehabilitation Sciences, KU Leuven, Leuven, Belgium; 5 National Jewish Medical and Research Center, Denver, CO USA; 6 Channing Division of Network Medicine, Department of Medicine, Brigham and Women’s Hospital and Harvard Medical School, Boston, MA USA; 7 University of Utah, Salt Lake City, Utah USA; 8 Department of Development and Regeneration, KU Leuven, Leuven, Belgium

**Keywords:** COPD, Computed tomography, Maximal expiratory flow-volume curve, Principal component analysis

## Abstract

**Background:**

Parameters from maximal expiratory flow-volume curves (MEFVC) have been linked to CT-based parameters of COPD. However, the association between MEFVC shape and phenotypes like emphysema, small airways disease (SAD) and bronchial wall thickening (BWT) has not been investigated.

**Research question:**

We analyzed if the shape of MEFVC can be linked to CT-determined emphysema, SAD and BWT in a large cohort of COPDGene participants.

**Study design and methods:**

In the COPDGene cohort, we used principal component analysis (PCA) to extract patterns from MEFVC shape and performed multiple linear regression to assess the association of these patterns with CT parameters over the COPD spectrum, in mild and moderate-severe COPD.

**Results:**

Over the entire spectrum, in mild and moderate-severe COPD, principal components of MEFVC were important predictors for the continuous CT parameters. Their contribution to the prediction of emphysema diminished when classical pulmonary function test parameters were added. For SAD, the components remained very strong predictors. The adjusted R^2^ was higher in moderate-severe COPD, while in mild COPD, the adjusted R^2^ for all CT outcomes was low; 0.28 for emphysema, 0.21 for SAD and 0.19 for BWT.

**Interpretation:**

The shape of the maximal expiratory flow-volume curve as analyzed with PCA is not an appropriate screening tool for early disease phenotypes identified by CT scan. However, it contributes to assessing emphysema and SAD in moderate-severe COPD.

**Supplementary Information:**

The online version contains supplementary material available at 10.1186/s12931-023-02318-4.

## Introduction

Chronic obstructive pulmonary disease (COPD) is often diagnosed after significant loss of lung function, as symptoms can remain mild to absent, and are often neglected by patients in the early disease stages. COPD is presumed to start as a smoldering disease, with small airways and parenchymal damage accumulating for many years without being noticed by patients or physicians [1, 2]. The ability to identify COPD in the early stage is key in the appropriate management of the disease aimed at improving patient outcomes, as well as reducing overall costs [3]. Spirometry is currently put forward as the most appropriate diagnostic tool, as it is non-invasive, easy to perform, and implementable at low cost. The spirometry diagnosis of COPD is based on a post-bronchodilator forced expiratory volume in one second/forced vital capacity (FEV1/FVC) ratio below the lower limit of the reference population in a clinical context of exposure to noxious particles [4].

A reduced forced expiratory flow between 25 and 75% of FVC (FEF25-75) has been proposed as a sign of small airways disease, in smokers only at risk of developing COPD [5, 6]. Moreover, recent large population studies in smoking individuals demonstrate that early pathological changes visualized on CT may also occur in subjects with ‘normal’ spirometry [7]. Normal, if not only defined by the FEV1/FVC ratio, is outlined by spirometry parameters varying within the range of a healthy non-smoking reference group [8, 9]. Even within the range of normality, the shape or contour of the maximal expiratory flow-volume curve (MEFVC) has been of continuous interest [10]. The concavity of the curve, often referred to as the kink, has been associated with emphysema and attributed to airway collapse and loss of elastic recoil [11]. Topalovic et al*.* proposed the angle of collapse of MEFVC to quantify airway collapse and detecting CT-defined emphysema in heavy smokers [12]. Dominelli et al*.* quantified the shape of MEFVC with the slope ratio index [13]. Bhatt et al*.* later proposed the parameter D which describes lung volume as an exponential function of time and the peak index, modeling the number of peaks adjusted for lung size [14, 15]. An overview of all indices can be found in the comprehensive review by Hoesterey et al. [16]. In this review, it has been postulated that further analysis on the shape of MEFVC yields the potential to discover parameters that can help detect early airway obstruction [16].

In a large subgroup of the Genetic Epidemiology of COPD study (COPDGene), we used principal component analysis (PCA) to comprehensively characterize the shape of MEFVC and linked the PCA components to CT-based parameters in subjects with mild and moderate-severe airflow obstruction.

## Study design and methods

### Study subjects

We used subjects enrolled in the COPDGene study, which is a large US-based multicenter study including current and former smokers aged 45–80 years (n = 10,198) with at least ten pack-years. Details of the study design have been reported previously [17]. The study was approved by local Institutional Review boards at each of the 21 clinical centers and all subjects provided written documentation of informed consent. The available data included raw spirometry and CT imaging data. For this analysis, we split the subjects on stages of the Global Initiative for Chronic Obstructive Lung Diseases (GOLD) guidelines according to FEV1, FVC and FEV1/FVC. GOLD I subjects belonged to the mild stage group while GOLD II-III-IV subjects belonged to the moderate-severe stage group.

### Spirometry and CT imaging data

Using a standardized protocol [18] and spirometer (NDD EasyOne Spirometer), 9841 participants performed spirometry. Expiratory flow-volume curves and volume-time curves were available. CT scans were obtained at total lung capacity (TLC) and at the end of normal expiration (functional residual capacity, FRC) using multi-detector CT scanners. CT densitometry was used to define the presence of emphysema and Small Airways Disease (SAD). Both %emphysema and %gas-trapping were computed using parametric response mapping (PRM) to identify the extent of emphysema (PRM^emph^) and functional small airways disease (PRM^fSAD^) based on CT scans at TLC and FRC simultaneously [19, 20]. Bronchial Wall Thickening (BWT) was assessed by airway wall thickness at an internal perimeter of 10 mm (Pi10). Pi10 was calculated by fitting a linear regression model on all airways of different internal perimeters with the square root of the wall area as dependent variable and perimeter as independent variable. Quantitative parameters of these scans were extracted using Thirona software.

### Shape analysis

To focus purely on the shape of MEFVC, we scaled each curve in both axes by 1/FVC for each subject to normalize on FVC and to preserve the shape of the curves. To perform a shape analysis, we applied PCA on the curves (flow over volume datapoints) to extract the most dominant patterns ordered according to the proportion in shape variance they explain. Each MEFVC could then be accurately approximated as a linear combination of these principal components (PC) or patterns with the coefficients describing how much each pattern contributed to the shape of the MEFVC. We computed these coefficients for all subjects in the dataset and linked these to the continuous CT parameters. We denoted the PCs following their order, e.g., the first PC was denoted as PC1. A more extensive description of the PCA computation can be found in the online supplement.

### CT-based phenotypes

With the quantitative CT (QCT) values, we defined the presence of emphysema, SAD and BWT using the upper limit of normal (95^th^ percentile, ULN) cut-offs based on never-smoked normal control subjects in the COPDGene dataset, 107 of such control subjects were enrolled in Phase 1. Based on these cut-offs, we defined eight CT-based phenotypes according to the presence of emphysema and/or SAD and/or BWT. For notation of the phenotypes, emphysema, SAD and BWT were denoted as E, S and B, respectively. A dash was used in the absence of a disease. An overview of all notations can be found in Table 1. We compared the PRM cut-offs with the ULN cut-offs when the %voxels < − 950 Hounsfield Units (Hu) and %voxels < − 856 Hu definitions for emphysema and SAD, respectively, were used on the same never-smoked normal subjects.

**Table 1:** Table with the used abbreviations of CT-defined phenotypes

	Emphysema	Small airways disease	Bronchial wall thickening
---	–	–	–
E--	✓	–	–
-S-	–	✓	–
--B	–	–	✓
ES-	✓	✓	–
-SB	–	✓	✓
E-B	✓	–	✓
ESB	✓	✓	✓

B, bronchial wall thickening; E, emphysema; S, small airways disease

### Data and statistical analysis

We performed descriptive statistics on demographic, spirometric and CT variables per GOLD stage and per CT-based phenotype. The data is presented as no. (%) or median [Q1-Q3 interquartile range]. Multiple linear regression was used to assess the independent effect of each component in predicting PRM^emph^, PRM^fSAD^ and Pi10 with adjustment for age, sex, height, weight and pack-years. Standard spirometric parameters (FEV1, FVC, FEV1/FVC, PEF, and FEF25-75) were then added and the standardized β coefficients of the model were used to assess the importance of each predictor. We used the adjusted R^2^, the coefficient of determination, to evaluate the goodness-of-fit of the models. Regression analysis was done over the entire spectrum, in mild COPD (GOLD I) and moderate-severe COPD (GOLD II-III-IV). We compared the principal components with existing MEFVC parameters: angle of collapse [21], area under the forced expiratory flow-volume loop [22], obstructive index [23] and peak index [15]. Statistical analysis was conducted using Python 3 (Python Software Foundation) with the scientific and statistical packages SciPy and StatsModels (open source, scipy.org and statsmodels.org), significance level was set at 0.05.

## Results

### Population characteristics

Of the 9841 patients that performed spirometry, 9207 (93.6%) had acceptable flow-volume loops according to the American Thoracic Society (ATS)/European Respiratory Society (ERS) guidelines [18]. Subjects with Preserved Ratio Impaired Spirometry (PRISm, FEV1/FVC > = 0.7 but FEV1 < 80%, n = 1055) were not considered, since other disease factors such as thoracic wall restriction or cardiac disease, being more prevalent in this subgroup, would influence our findings [24, 25]. Ultimately, 6302 subjects were used for the analysis. The flow of the eligible subjects for this analysis is described in Additional file 1: Figure S1. The characteristics of the remaining participants per GOLD stage are reported in Table 1. Sixty-seven of 107 never-smoked control subjects had both spirometry and CT data available. In the 6302 subjects used for the analysis, 67 were non-smokers and 6235 were ever smokers. Of those 6235 ever smokers, 3214 were former smokers and 3021 were current smokers.

**Table 2:** Characteristics per GOLD stage

		Never-smoked normal	GOLD 0	GOLD I	GOLD II	GOLD III	GOLD IV
N		67	2842	567	1483	892	451
Age (years)		59.5 [53.9–66.8]	55.6 [50.2–63.0]	61.9 [55.0–68.9]	62.6 [55.9–69.4]	65.2 [58.7–71.1]	64.4 [59.2–69.8]
Sex (%Males)		23 (34%)	1434 (50%)	323 (57%)	782 (53%)	522 (59%)	269 (60%)
Weight (kg)		81.0 [66.0–88.3]	82.7 [71.1–95.2]	77.6 [67.0–87.0]	82.0 [69.2–94.8]	80.0 [65.4–94.0]	72.2 [60.2–84.6]
Height (cm)		166.4 [159.8–173.4]	170.0 [163.0–177.0]	170.0 [162.6–177.8]	170.0 [162.9–176.6]	169.9 [162.6–177.0]	170.2 [163.0–176.6]
BMI (kg/m2)		27.5 [24.5–31.7]	28.3 [25.0–32.5]	26.5 [23.7–29.8]	27.9 [24.5–32.1]	27.1 [23.6–31.7]	24.8 [21.6–28.8]
Pack-years		0.0 [0.0–0.0]	34.2 [22.6–46.3]	40.8 [30.0–54.5]	45.5 [33.6–64.5]	49.5 [38.0–69.0]	49.5 [38.0–74.5]
FEV1 (L)		2.8 [2.3–3.4]	2.8 [2.4–3.3]	2.6 [2.2–3.1]	1.8 [1.5–2.2]	1.1 [0.9–1.3]	0.7 [0.5–0.8]
FEV1 (%Pred)		103.4 [93.8–115.6]	95.9 [88.5–104.4]	88.3 [84.2–95.2]	65.6 [57.7–72.0]	40.5 [35.4–45.0]	23.6 [19.0–26.7]
FVC (L)		3.6 [2.9–4.3]	3.6 [3.0–4.3]	4.1 [3.4–4.8]	3.1 [2.6–3.8]	2.6 [2.1–3.2]	2.1 [1.7–2.6]
FVC (%Pred)		98.5 [90.5–109.3]	95.5 [88.4–104.1]	105.3 [98.5–112.7]	85.2 [77.5–93.2]	70.7 [62.3–79.0]	55.5 [46.9–65.7]
FEV1/FVC		0.8 [0.8–0.8]	0.8 [0.7–0.8]	0.7 [0.6–0.7]	0.6 [0.5–0.7]	0.4 [0.4–0.5]	0.3 [0.3–0.4]
PEF (L/s)		7.8 [6.5–9.2]	7.4 [6.2–9.0]	6.9 [5.6–8.7]	5.2 [4.2–6.5]	3.4 [2.7–4.3]	2.3 [1.8–2.9]
FEF25-75 (L/s)		3.1 [2.3–3.6]	2.6 [2.0–3.3]	1.3 [0.9–1.6]	0.7 [0.5–1.0]	0.4 [0.3–0.4]	0.2 [0.2–0.3]
%Emphysema		0.2 [0.0–0.5]	0.2 [0.1–0.8]	1.5 [0.4–4.3]	2.7 [0.7–8.2]	13.6 [4.8–24.8]	27.0 [16.6–39.2]
%Gas Trapping		6.5 [4.3–10.0]	6.6 [3.1–11.2]	13.9 [8.0–21.5]	19.6 [11.8–28.5]	33.0 [25.0–39.5]	36.9 [31.7–43.5]
Pi10 (mm)		1.7 [1.5–1.9]	1.9 [1.7–2.2]	2.0 [1.8–2.4]	2.5 [2.2–2.9]	2.8 [2.5–3.2]	2.9 [2.5–3.2]

Data are presented as median [Q1-Q3 interquartile range] and no. (%)

BMI, body mass index; FEF25-75, mean forced expiratory flow between 25 and 75% of FVC; FEV1, forced expiratory volume in 1 s; FVC, forced vital capacity; GOLD, Global Initiative for Chronic Obstructive Lung Diseases; PEF, peak expiratory flow; Pi10, internal perimeter of 10 mm

### Principal components

**Fig. 1:**
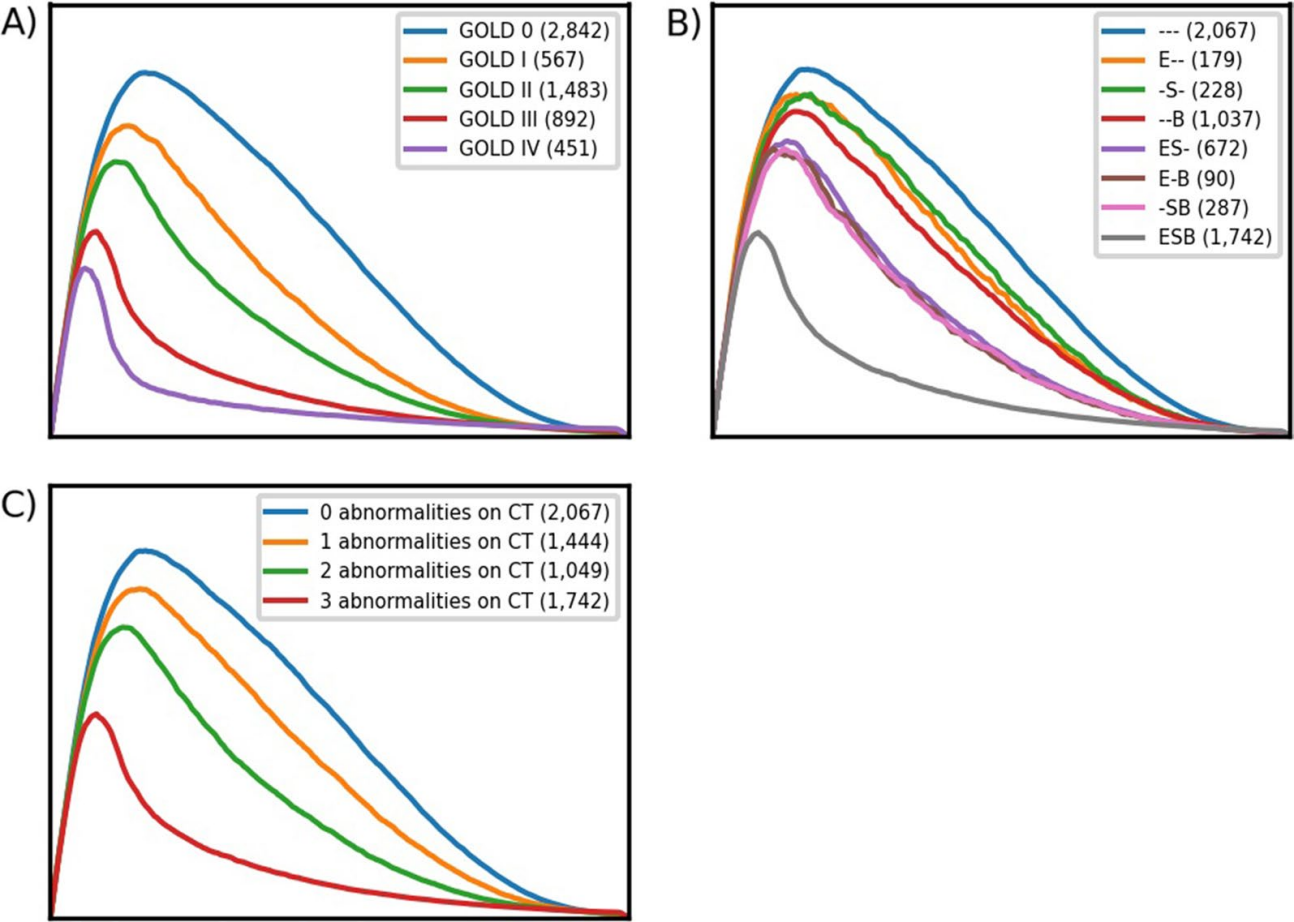
The mean MEFVC shapes per GOLD stage in panel **A**, the mean MEFVC shapes per CT-based phenotype in panel **B** and the mean MEFVC shapes per number of abnormalities on CT in panel** C**. B, bronchial wall thickening; E, emphysema; GOLD, Global Initiative for Chronic Obstructive Lung Disease; MEFVC, maximal expiratory flow-volume curve; S, small airways disease

**Fig. 2:**
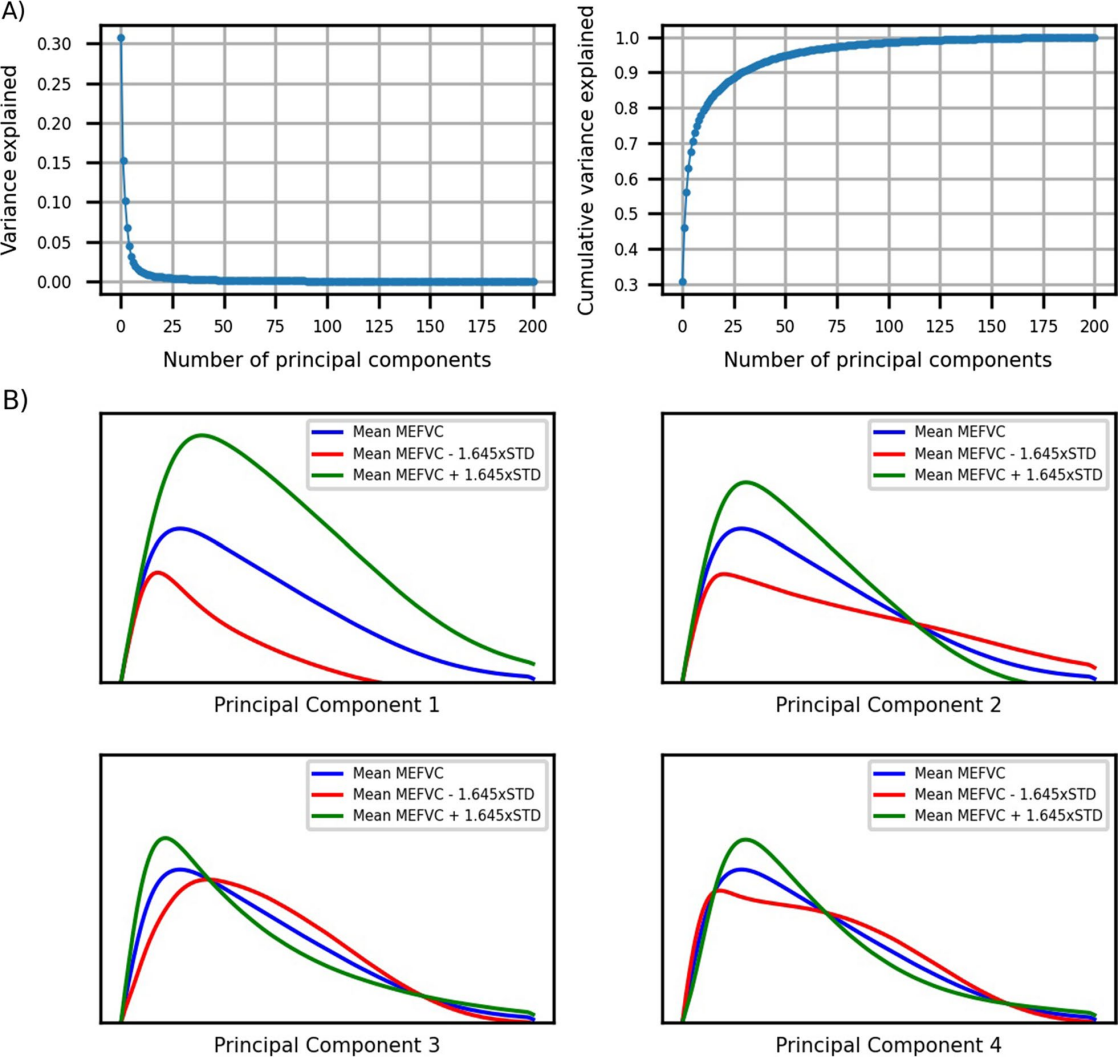
**A** Variance explained by the principal components on the left, cumulative variance explained by the principal components on the right. **B** influence of the first four principal components visualized. The blue curve is the overall mean MEFVC shape. The green curve illustrates the influence of each principal component when the coefficient is increased to the 95^th^ percentile, the red curve when the coefficient is decreased to the 5^th^ percentile. Component 1 influences PEF and the descending limb without altering the angle of collapse or concavity. Component 2 pivots the descending limb around a fixed point, thereby also influencing PEF. Component 3 and 4 mainly model concavity in MEFVC

The mean standardized flow-volume curves per GOLD stage are visualized in Fig. 1. The curves were sampled at 200 equidistant points resulting in 200 principal components (full decomposition) with the first ten explaining 78% of the variance in MEFVC shape (Fig. 2A). With the first 100 components, 98.4% of the variance could be explained. To visualize the influence of the components on MEFVC, we depicted a − 45 to + 45 percent change (5^th^ to 95^th^ percentile) of the four most dominant components as compared to the overall mean MEFVC of the population (Fig. 2B). We visually assessed the influence of each of these four components on the MEFVC: PC1 influences PEF and the descending limb without altering the angle of collapse or concavity. PC2 pivots the descending limb around a fixed point, thereby also influencing PEF. PC3 and PC4 mainly model concavity in MEFVC. The remainder of the analyses were done with the first four principal components since more components did not improve the model fits (adjusted R^2^) in the following analyses.

### ***Multivariate analysis on PRM***^***emph***^

When only considering principal components and adjusting for age, sex, height, weight and pack-years, PC1 (β = − 4.9, *P* < 0.001), PC2 (β = − 4.3, *P* < 0.001) and PC3 (β = − 1.4, *P* < 0.001) were significant predictors in the entire COPDGene population. Adjusted R^2^ was 0.50. In mild COPD, PC1 (β = − 1.6, *P* < 0.001), PC2 (β = − 1.4, *P* < 0.001) and PC3 (β = 0.7, *P* = 0.001) were significant predictors and adjusted R^2^ was 0.17. In moderate-severe COPD, PC1 (β = − 2.5, *P* < 0.001), PC2 (β = − 7.3, *P* < 0.001), PC3 (β = 2.3, *P* < 0.001) and PC4 (β = 0.9, *P* = 0.001) were significant predictors with an adjusted R^2^ of 0.50. Full results can be found in Table 3.

**Table 3:** Multivariate analysis for PRM^emph^

	Entire spectrum (n = 6302)				Mild COPD (n = 567)				Moderate−severe COPD (n = 2826)			
	Adjusted R^2^ = 0.50				Adjusted R^2^ = 0.17				Adjusted R^2^ = 0.50			
	β		*P-*value		β	*P*-value			β		*P*-value	
Age	1.32		< 0.001		1.08	< 0.001			1.40		< 0.001	
Sex	− 0.58		< 0.001		− 0.70	0.01			− 0.06		0.80	
Height	0.63		< 0.001		0.38	0.16			1.71		< 0.001	
Weight	− 1.80		< 0.001		− 0.91	< 0.001			− 3.18		< 0.001	
Pack-years	0.17		0.09		0.35	0.05			− 0.12		0.51	
PC1	− 4.87		< 0.001		− 1.55	< 0.001			− 2.49		< 0.001	
PC2	− 4.34		< 0.001		− 1.42	< 0.001			− 7.28		< 0.001	
PC3	− 0.05		0.61		0.70	0.001			2.25		< 0.001	
PC4	− 1.37		< 0.001		0.01	0.97			0.90		0.001	

COPD, Chronic Obstructive Pulmonary Disease; PC, principal component

When adding standard spirometric parameters tests to the model, the principal components, PC1 (β = 0.7, *P* < 0.01), PC2 (β = 0.7, *P* < 0.01) and PC3 (β = − 0.4, *P* < 0.01) were still significantly associated in the entire COPDGene population. The adjusted R^2^ was 0.67. In mild COPD, adjusted R^2^ was 0.28 with FEV1 the dominant predictor (β = − 10.7, *P* < 0.001). PC4 was a significant predictor (β = − 0.5, *P* = 0.04). In moderate-severe COPD, adjusted R^2^ was 0.59 with FEV1/FVC the dominant predictor (β = − 11.3, *P* < 0.001), none of the components were significantly associated. Full results can be found in Additional file 1: Table S1.

### ***Multivariate analysis on PRM***^***fSAD***^

Adjusting for age, sex, height, weight and pack-years and only considering principal components, PC1 (β = − 7.1, *P* < 0.001), PC2 (β = − 5.1, *P* < 0.001), PC3 (β = 1.1, *P* < 0.001) and PC4 (β = − 0.7, *P* < 0.001) were significant predictors. Adjusted R^2^ was 0.60. In mild COPD, PC1 (β = − 2.3, *P* < 0.001), PC2 (β = − 2.6, *P* < 0.001) and PC3 (β = 0.9, *P* = 0.03) were significant predictors while adjusted R^2^ was 0.2. In moderate-severe COPD, PC1 (β = − 2.5, *P* < 0.001), PC2 (β = − 6.8, *P* < 0.001), PC3 (β = 2.4, *P* < 0.001) and PC4 (β = 0.7, *P* = 0.01) were significant predictors with an adjusted R^2^ of 0.48. Full results can be found in Table 4.

**Table 4:** Multivariate analysis for PRM^fSAD^

	Entire spectrum (n = 6302)		Mild COPD (n = 567)		Moderate−severe COPD (n = 2826)	
	Adjusted R^2^ = 0.60		Adjusted R^2^ = 0.20		Adjusted R^2^ = 0.48	
	β	*P*-value	β	*P*-value	β	*P*-value
Age	2.73	< 0.001	3.33	< 0.001	2.13	< 0.001
Sex	− 1.24	< 0.001	− 1.12	0.03	− 0.96	< 0.001
Height	0.63	< 0.001	1.21	0.03	0.70	0.01
Weight	− 1.53	< 0.001	− 1.68	< 0.001	− 1.93	< 0.001
Pack-years	0.61	< 0.001	0.30	0.40	0.56	0.002
PC1	− 7.14	< 0.001	− 2.35	< 0.001	− 2.54	< 0.001
PC2	− 5.10	< 0.001	− 2.63	< 0.001	− 6.79	< 0.001
PC3	1.11	< 0.001	0.88	0.03	2.68	< 0.001
PC4	− 0.66	< 0.001	− 0.52	0.17	0.69	0.01

COPD, Chronic Obstructive Pulmonary Disease; PC, principal component

When adding standard spirometric parameters to the model, the principal components PC1 (β = − 2.2, *P* < 0.001), PC2 (β = − 1.5, *P* < 0.001) and PC3 (β = 0.7, *P* < 0.001) were still significant predictors. Adjusted R^2^ over the entire population was 0.65. In mild COPD, PC1, PC2 and PC4 were significant predictors (β = − 1.4, − 2.4 and − 1.1, *P* = 0.03, *P* = 0.004 and *P* = 0.02). In moderate-severe COPD, PC1, PC2 and PC3 were significant predictors (β = − 1.4, − 5.9 and 2.5, *P* < 0.001, *P* < 0.001 and *P* < 0.001) with an adjusted R^2^ of 0.49. Full results can be found in Additional file 1: Table S2.

### Multivariate analysis on BWT

Only considering principal components and after adjusting for age, sex, height, weight and pack-years, all four PCs were significant predictors over the entire spectrum (β = − 0.35, β = − 0.19, β = 0.045 and β = 0.02, *P* < 0.001, respectively) and adjusted R^2^ was 0.39. In mild COPD, PC1 and PC2 were significant predictors (β = − 0.06 and β = − 0.06, *P* = 0.04 and *P* = 0.1, respectively) with adjusted R^2^ 0.14 while in moderate-severe COPD, PC2, PC3 and PC4 were significant predictors (β = − 0.13, β = − 0.03 and β = − 0.05, *P* < 0.001, *P* = 0.03 and *P* = 0.001, respectively) with an adjusted R^2^ of 0.16. Full results can be found in Additional file 1: Table S3.

Again, the principal components were of little benefit on top of classical pulmonary function variables with an adjusted R^2^ over the entire population of 0.48 and with PC1, PC3 and P4 as significant predictors (β = − 0.04, β = 0.04 and β = 0.02, *P* = 0.02, *P* < 0.001 and *P* = 0.004, respectively). In mild COPD, adjusted R^2^ was 0.19 and no component was a significant predictor. In moderate-severe COPD, adjusted R^2^ was 0.23 with PC2 and PC3 significant predictors (β = − 0.11 and β = 0.05, *P* = 0.001 and *P* = 0.02, respectively). Full results can be found in Additional file 1: Table S4.

### CT-Phenotypes

**Fig. 3:**
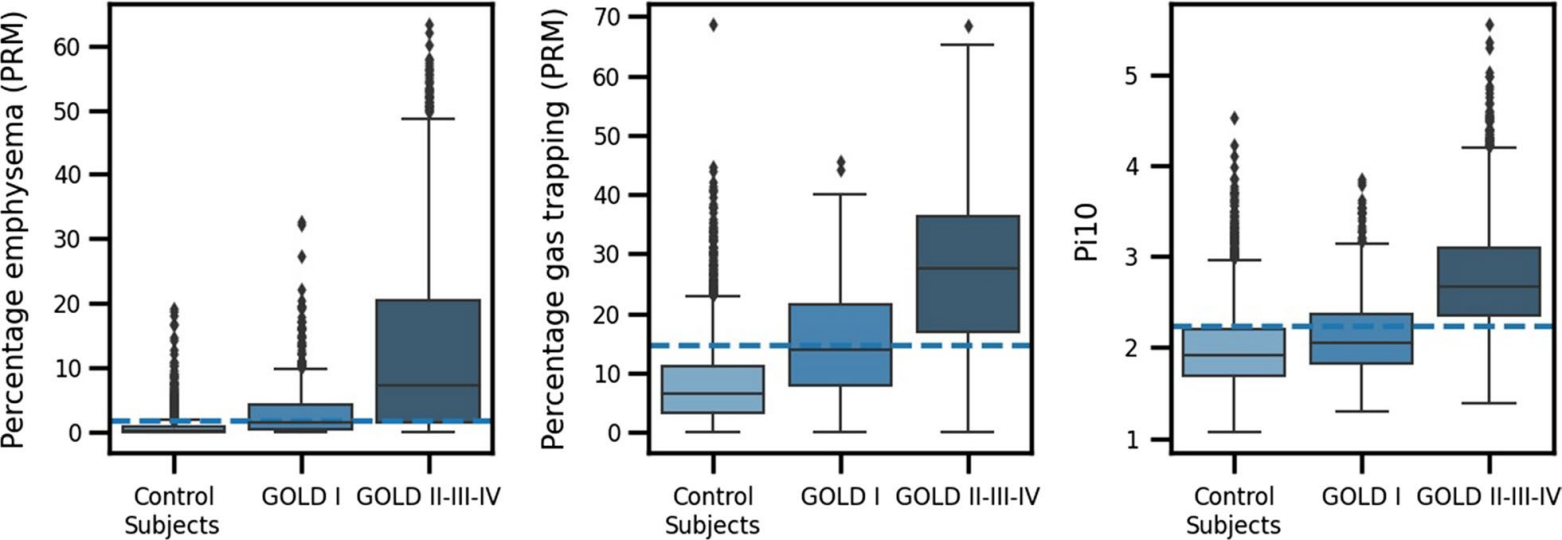
Percentage emphysema (PRM^emph^), percentage gas trapping (PRM^fSAD^) and bronchial wall thickening (Pi10) per group (never-smoked normal control subjects; mild COPD (GOLD I); moderate-severe COPD (GOLD II-III-IV)). The cut-offs (dashed lines) are determined by using the 95^th^ percentile (upper limit of normal) on the control subjects

We determined 1.7% for PRM^emph^, 14.7% for PRM^fSAD^ and 2.2 mm for Pi10 (Fig. 3) as the upper limit of normal (ULN) in a cohort of never-smoked normal controls (n = 67) and considered them as cut-offs for the presence of CT-based abnormalities. Figure 1B shows the mean MEFVC per CT-based phenotype and Fig. 1C the mean MEFVC per number of abnormalities as seen on CT. The characteristics of the subjects per CT-based phenotype are reported in Additional file 1: Table S5. The cut-offs for emphysema and SAD were 5.8 and 18.6% when the classic %voxels < − 950 and %voxels < − 856 definitions for emphysema and SAD were used.

### Comparison with other MEFVC-derived parameters

Adjusted R^2^ for each parameter per CT outcome and subgroup can be found in Additional file 1: Table S6. Compared to other MEFVC-derived parameters, the principal components provided a better fit for PRM^emph^ and PRM^SAD^. For Pi10, area under the forced expiratory flow-volume loop was the superior parameter. Overall, the classical pulmonary function parameters were superior.

## Discussion

In 6302 subjects in the COPDGene study, we used principal component analysis (PCA) to extract dominant patterns from the shapes of the MEFVC and explored their association with continuous CT-based parameters and eight CT-defined phenotypes based on cut-offs for emphysema, small airways disease and bronchial wall thickening. The advantage of this PCA analysis is that no hand-engineered features were required to analyze the MEFVC and the large collection of curves was fully exploited in extracting potential patterns. When compared with existing hand-engineered features, the principal components were superior for emphysema and small airways disease and closely matched the area under the MEFVC for bronchial wall thickening.

We found that a small number of components were sufficient to model a large proportion of the variance in shape of MEFVC. Multivariate analysis for the first four principal components showed that 49, 60 and 39 percent of the variance could be explained for emphysema (PRM^emph^), small airways disease (PRM^fSAD^) and bronchial wall thickening (Pi10), respectively. However, when adding classical pulmonary function tests (FEV1, FVC, FEV1/FVC, PEF and FEF25-75) to the models, independent contributions of the principal components were strongly reduced because of high intra-correlations (Additional file 1: Table S7). For emphysema (PRM^emph^), shape-derived components PC1, PC2 and PC3 were still independent contributors. For small airways disease (PRM^fSAD^) in mild COPD, PC2 was the third important predictor, whilst it became the most important predictor in moderate-severe COPD for which FEV1, FVC and FEV1/FVC were no longer significant. These findings highlight the impact of small airways disease on PEF, particularly in more advanced disease stages. For bronchial wall thickening, the fit of the regression model was generally very low, indicating that presence of abnormal thickening of the larger airway bronchial walls, did not profoundly affect the shape of the MEFVC.

Interestingly, in multinomial logistic regression modelling the number of CT disease abnormalities present on CT rather the type of CT abnormalities (E, SAD, BWT), the pseudo R^2^ was negative and not significant for mild COPD, indicating that in the mild disease stage, any CT abnormalities are unlikely to be detected by the shape of MEFVC or even the standard lung function parameters. Hence, the current data suggest that our initial hypothesis should be rejected, and that early disease processes as identified on CT cannot be predicted by parameters isolated from the relative form of the maximal expiratory flow-volume curve. In particular, FEF25-75, as surrogate marker of small airways disease on spirometry was not predictive in the mild COPD subgroup. It raises the question if in patients with normal spirometry, risk behavior and chronic respiratory symptoms may point to the need of a CT scan, as suggested by Celli et al. [26].

By using the upper limit of normal on the 67 never-smoked normal control subjects in this cohort, we obtained cut-offs for abnormal values of the CT outcomes. With these cut-off values, most of the patients in the mild COPD subgroup had PRM^emph^ and PRM^fSAD^ values within the normal range (Fig. 3). It also demonstrates that mild airflow limitation as diagnosed by an FEV1/FVC below 0.7, can present with CT scans within normal limits. In these individuals, early airway pathology in terminal or respiratory bronchioles may still be present as this is beyond the resolution of conventional CT [27]. An alternative explanation may come from the initial lung function values determined by lung growth, which may result in a lower FEV1/FVC ratio and FEV1 without true pathology on CT.

We normalized the MEFVC curve for FVC to adjust for lung volume and hence also anthropometry and age, and to maximally visualize the changes in shape across the different phenotypes. Next, we calculated the mean MEFVC shape per GOLD stage and per CT-based phenotype. The area under the curve decreases and the concavity or so-called kink in the curve increases as lung function deteriorates. For the CT-based phenotypes, the mean shapes are similar for the phenotypes with only one abnormality [--B, -S-, E--], while for those with two abnormalities [-SB, E-B, ES-], the concavity is larger and the area under the curve smaller, which is to be expected as these are the subjects in the higher GOLD stages. Subjects showing evidence of three abnormalities on CT have the largest concavity and the smallest area under the curve on average. Overall, our findings indicate that concavity of the flow-volume loop is linked to more advanced COPD in which emphysema, but also other radiological phenotypes co-occur.

## Interpretation

Our analysis demonstrates that the shape of the maximal expiratory flow-volume curve is not an appropriate screening tool for early disease phenotypes identified by CT scan since neither the principal components and classical pulmonary function parameters were linked with emphysema, small airways disease or bronchial wall thickening as seen on CT. In moderate-severe airflow obstruction (GOLD II-III-IV) the concavity of the curve is mainly related to the presence of emphysema in a combined phenotype with small airways disease, with MEFVC shape parameters having a limited but statistically significant association with CT defined pathologies.

## Supplementary Information

**Additional file 1: Table S1.** Multivariate analysis for PRM^emph^ with pulmonary function test parameters. **Table S2.** Multivariate analysis for PRM^fSAD^ with pulmonary function test parameters.**Table S3.** Multivariate analysis for Pi10. **Table S4.** Multivariate analysis for Pi10 with pulmonary function test parameters. **Table S5.** Characteristics per CT-based phenotype. **Table S6.** Linear regression adjusted R^2^s for different parameters derived from maximal expiratory flow-volume curves for emphysema, small airways disease and bronchial wall thickening (PRM^emph^, PRM^f^^SAD^, Pi10 on CT) per subgroup. **Table S7.** Pearson correlation coefficients between the first four principal components and the classical pulmonary function parameters FEV1, FVC, FEV1/FVC, FEF25-75, PEF. **Figure S1.** Flow of eligible subjects for this study. CT, computed tomography; COPDGene, Genetic Epidemiology of COPD; PRISm, preserved ratio impaired spirometry.

## Data Availability

The dataset supporting the conclusions of this article is available by request through the COPDGene investigators (https://copdgene.org/).

## Abbreviations

ATS: American Thoracic Society
BWT: Bronchial wall thickening
COPD: Chronic obstructive pulmonary disease
CT: Computed tomography
emph: Emphysema
ERS: European Respiratory Society
FEF: Forced expiratory flow
FEV1: Forced expiratory volume in 1 second
FRC: Functional residual capacity
fSAD: Functional Small Airways Disease
FVC: Forced vital capacity
GOLD: Global Initiative for Chronic Obstructive Lung Disease
Hu: Hounsfield unit
MEFVC: Maximal Expiratory Flow-volume Curve
PC: Principal component
PCA: Principal component analysis
PEF: Peak expiratory flow
Pi10: Airway wall thickness at an internal perimeter of 10 mm
PRISm: Preserved ratio impaired spirometry
PRM: Parametric response mapping
SAD: Small Airways Disease
TLC: Total lung capacity
ULN: Upper limit of normal
