# Supplementary material for: Principal component analysis of flow-volume curves in COPDGene to link spirometry with phenotypes of COPD
Source: Respir Res. 2023 Jan 19;24:20. doi: 10.1186/s12931-023-02318-4 (PMC9854102; doi:10.1186/s12931-023-02318-4)
Supplement: Supplementary file 1 — Additional file 1: Table S1. Multivariate analysis for PRMemph with pulmonary function test parameters. Table S2. Multivariate analysis for PRMfSAD with pulmonary function test parameters.Table S3. Multivariate analysis for Pi10. Table S4. Multivariate analysis for Pi10 with pulmonary function test parameters. Table S5. Characteristics per CT-based phenotype. Table S6. Linear regression adjusted R2s for different parameters derived from maximal expiratory flow-volume curves for emphysema, small airways disease and bronchial wall thickening (PRMemph, PRMfSAD, Pi10 on CT) per subgroup. Table S7. Pearson correlation coefficients between the first four principal components and the classical pulmonary function parameters FEV1, FVC, FEV1/FVC, FEF25-75, PEF. Figure S1. Flow of eligible subjects for this study. CT, computed tomography; COPDGene, Genetic Epidemiology of COPD; PRISm, preserved ratio impaired spirometry. [file 12931_2023_2318_MOESM1_ESM.docx]

**Additional text**

## Shape analysis

Each maximal expiratory flow-volume curve (MEFVC) is represented by a single vector describing the flow at each volume point sampled every 60 ms. We scaled each curve in both axes by 1/FVC for each subject to normalize on FVC and to preserve the shape of the curves. Because the length of this vector is different for each subject, we resampled the MEFVC for each subject to obtain vectors of length 200. For each of the 6302 analyzed subjects, we then had a normalized MEFVC represented by a vector of length 200.

A matrix of size 6302x200 is obtained with the normalized MEFVCs of all subjects. On this, the singular value decomposition is applied the perform principal component analysis. This decomposition extracts the principal components as seen in Figure 2 while for each subject the coefficients are also computed. The coefficient of a component for a subject indicates the influence of that component on the shape of the MEFVC for that subject.

**Additional tables**

|  | **Entire spectrum (n=6,302)** | | **Mild COPD (n=567)** | | **Moderate-severe COPD (n=2,826)** | |
| --- | --- | --- | --- | --- | --- | --- |
|  | Adjusted R^2^ = 0.67 | | Adjusted R^2^ = 0.28 | | Adjusted R^2^ = 0.59 | |
|  | β | *P*-value | β | *P*-value | β | *P*-value |
| **Age** | 0.58 | <0.001 | 0.99 | <0.001 | 0.97 | <0.001 |
| **Sex** | 0.09 | 0.47 | -0.29 | 0.28 | 0.20 | 0.41 |
| **Height** | 0.87 | <0.001 | 0.17 | 0.60 | 1.72 | <0.001 |
| **Weight** | -1.58 | <0.001 | -0.80 | <0.001 | -2.83 | <0.001 |
| **Pack-years** | -0.08 | 0.34 | 0.31 | 0.06 | -0.15 | 0.36 |
| **FEV1** | -2.38 | <0.001 | -10.70 | <0.001 | -3.22 | 0.01 |
| **FVC** | -0.39 | 0.33 | 8.55 | <0.001 | -0.20 | 0.78 |
| **FEV1/FVC** | -11.66 | <0.001 | -0.03 | 0.97 | -11.26 | <0.001 |
| **PEF** | 0.10 | 0.76 | 1.25 | 0.02 | 0.02 | 0.98 |
| **FEF25-75** | 5.85 | <0.001 | 2.22 | <0.001 | 4.37 | <0.001 |
| **PC1** | 0.69 | <0.001 | -0.51 | 0.09 | 0.17 | 0.64 |
| **PC2** | 0.68 | <0.001 | -0.60 | 0.13 | 0.79 | 0.16 |
| **PC3** | -0.39 | <0.001 | -0.33 | 0.21 | 0.08 | 0.82 |
| **PC4** | -0.10 | 0.41 | -0.49 | 0.04 | 0.60 | 0.05 |

Table S1 – Multivariate analysis for PRM^emph^ with pulmonary function test parameters
Abbreviations: COPD, Chronic Obstructive Pulmonary Disease; FEF25-75, mean forced expiratory flow between 25% and 75% of FVC; FEV1, forced expiratory volume in 1 second; FVC, forced vital capacity; PC, principal component; PEF, peak expiratory flow; PRM^emph^, parametric response mapping for emphysema on CT.

|  | **Entire spectrum (n=6,302)** | | **Mild COPD (n=567)** | | **Moderate-severe COPD (n=2,826)** | |
| --- | --- | --- | --- | --- | --- | --- |
|  | Adjusted R^2^ = 0.65 | | Adjusted R^2^ = 0.21 | | Adjusted R^2^ = 0.49 | |
|  | β | *P*-value | β | *P*-value | β | *P*-value |
| **Age** | 2.03 | <0.001 | 3.01 | <0.001 | 1.74 | <0.001 |
| **Sex** | -1.22 | <0.001 | -1.08 | 0.06 | -1.56 | <0.001 |
| **Height** | 1.32 | <0.001 | 1.72 | 0.01 | 1.68 | <0.001 |
| **Weight** | -1.49 | <0.001 | -1.69 | <0.001 | -2.14 | <0.001 |
| **Pack-years** | 0.33 | <0.001 | 0.21 | 0.56 | 0.52 | <0.001 |
| **FEV1** | -0.65 | 0.45 | -10.00 | 0.06 | -0.54 | 0.67 |
| **FVC** | -1.28 | 0.01 | 6.54 | 0.18 | -0.50 | 0.52 |
| **FEV1/FVC** | -7.20 | <0.001 | 0.03 | 0.98 | 0.57 | 0.50 |
| **PEF** | -0.63 | 0.15 | 1.49 | 0.18 | -1.21 | 0.06 |
| **FEF25-75** | 1.93 | <0.001 | 1.95 | 0.06 | -1.20 | 0.04 |
| **PC1** | -2.18 | <0.001 | -1.42 | 0.03 | -1.40 | <0.001 |
| **PC2** | -1.49 | <0.001 | -2.39 | <0.001 | -5.92 | <0.001 |
| **PC3** | 0.66 | <0.001 | -0.08 | 0.89 | 2.47 | <0.001 |
| **PC4** | -0.14 | 0.35 | -1.13 | 0.02 | 0.41 | 0.22 |

Table S2 – Multivariate analysis for PRM^fSAD^ with pulmonary function test parameters
Abbreviations: COPD, Chronic Obstructive Pulmonary Disease; FEF25-75, mean forced expiratory flow between 25% and 75% of FVC; FEV1, forced expiratory volume in 1 second; FVC, forced vital capacity; PC, principal component; PEF, peak expiratory flow; PRM^fSAD^, parametric response mapping for functional small airways disease on CT.

|  | **Entire spectrum (n=6,302)** | | **Mild COPD (n=567)** | | **Moderate to severe COPD (n=2,826)** | |
| --- | --- | --- | --- | --- | --- | --- |
|  | Adjusted R^2^ = 0.39 | | Adjusted R^2^ = 0.14 | | Adjusted R^2^ = 0.16 | |
|  | β | *P*-value | β | *P*-value | β | *P*-value |
| **Age** | -0.12 | <0.001 | -0.11 | <0.001 | -0.10 | <0.001 |
| **Sex** | -0.05 | <0.001 | -0.07 | 0.01 | -0.06 | <0.001 |
| **Height** | -0.11 | <0.001 | -0.19 | <0.001 | -0.15 | <0.001 |
| **Weight** | 0.13 | <0.001 | 0.11 | <0.001 | 0.15 | <0.001 |
| **Pack-years** | 0.06 | <0.001 | 0.02 | 0.38 | 0.03 | <0.001 |
| **PC1** | -0.35 | <0.001 | -0.06 | 0.04 | -0.02 | 0.14 |
| **PC2** | -0.19 | <0.001 | -0.06 | 0.01 | -0.13 | <0.001 |
| **PC3** | 0.04 | <0.001 | -0.02 | 0.26 | -0.03 | 0.03 |
| **PC4** | 0.02 | <0.001 | 0.00 | 0.88 | -0.05 | <0.001 |

Table S3 – Multivariate analysis for Pi10
Abbreviations: COPD, Chronic Obstructive Pulmonary Disease; PC, principal component; Pi10, internal perimeter of 10 mm.

|  | **Entire spectrum (n=6,302)** | | **Mild COPD (n=567)** | | **Moderate to severe COPD (n=2,826)** | |
| --- | --- | --- | --- | --- | --- | --- |
|  | Adjusted R^2^ = 0.48 | | Adjusted R^2^ = 0.18 | | Adjusted R^2^ = 0.23 | |
|  | β | *P*-value | β | *P*-value | β | *P*-value |
| **Age** | -0.17 | <0.001 | -0.15 | <0.001 | -0.12 | <0.001 |
| **Sex** | -0.12 | <0.001 | -0.13 | <0.001 | -0.12 | <0.001 |
| **Height** | 0.00 | 0.81 | -0.06 | 0.08 | -0.05 | <0.001 |
| **Weight** | 0.11 | <0.001 | 0.09 | <0.001 | 0.14 | <0.001 |
| **Pack-years** | 0.03 | <0.001 | 0.01 | 0.67 | 0.03 | <0.001 |
| **FEV1** | -0.01 | 0.78 | 0.06 | 0.83 | -0.01 | 0.93 |
| **FVC** | -0.10 | <0.001 | -0.27 | 0.27 | -0.04 | 0.32 |
| **FEV1/FVC** | -0.05 | 0.10 | -0.13 | 0.08 | 0.11 | 0.02 |
| **PEF** | -0.22 | <0.001 | -0.11 | 0.06 | -0.23 | <0.001 |
| **FEF25-75** | -0.13 | <0.001 | 0.12 | 0.03 | 0.00 | 0.98 |
| **PC1** | -0.04 | 0.02 | 0.01 | 0.69 | -0.02 | 0.38 |
| **PC2** | -0.03 | 0.06 | <0.001 | 0.97 | -0.11 | <0.001 |
| **PC3** | 0.04 | <0.001 | <0.001 | 0.96 | 0.05 | 0.02 |
| **PC4** | 0.02 | <0.001 | 0.02 | 0.47 | -0.01 | 0.47 |

Table S4 – Multivariate analysis for Pi10 with pulmonary function test parameters
Abbreviations: COPD, Chronic Obstructive Pulmonary Disease; FEF25-75, mean forced expiratory flow between 25% and 75% of FVC; FEV1, forced expiratory volume in 1 second; FVC, forced vital capacity; PC, principal component; PEF, peak expiratory flow; Pi10, internal perimeter of 10 mm.

|  | **---** | **--B** | **-S-** | **-SB** | **E--** | **E-B** | **ES-** | **ESB** |
| --- | --- | --- | --- | --- | --- | --- | --- | --- |
| **N** | 2,067 | 1,037 | 228 | 287 | 179 | 90 | 672 | 1,742 |
| **Age (years)** | 56.4 [50.7-63.2] | 54.0 [49.3-60.4] | 62.5 [56.1-68.8] | 60.0 [54.0-66.6] | 61.2 [53.8-66.4] | 59.1 [52.1-66.5] | 65.7 [59.3-70.5] | 65.5 [58.8-71.3] |
| **Sex (%Males)** | 980 (47%) | 481 (46%) | 128 (56%) | 145 (51%) | 116 (65%) | 50 (56%) | 422 (63%) | 1031 (59%) |
| **Weight (kg)** | 83.0 [70.5-95.0] | 85.0 [74.2-98.9] | 78.0 [67.8-87.6] | 86.2 [73.4-104.0] | 79.0 [67.8-88.2] | 80.2 [70.9-94.8] | 75.8 [64.8-86.5] | 77.0 [65.0-89.9] |
| **Height (cm)** | 170.0 [162.6-176.5] | 168.0 [162.0-175.3] | 171.8 [163.4-178.1] | 169.5 [162.6-176.5] | 172.8 [165.9-177.9] | 170.0 [165.2-175.3] | 172.1 [165.0-179.0] | 170.0 [162.9-177.0] |
| **BMI (kg/m2)** | 28.3 [25.1-32.2] | 30.1 [26.0-34.3] | 26.3 [23.5-29.6] | 30.3 [25.3-35.0] | 26.1 [23.7-29.3] | 27.3 [25.2-31.3] | 25.6 [22.8-28.5] | 26.5 [23.1-30.7] |
| **Pack-years** | 33.4 [21.6-45.1] | 38.3 [27.0-51.0] | 37.0 [24.9-49.6] | 42.4 [32.5-60.0] | 37.6 [25.0-51.5] | 43.8 [33.0-59.9] | 43.5 [31.0-60.0] | 49.3 [37.8-70.5] |
| **FEV1 (L)** | 2.8 [2.4-3.3] | 2.3 [1.9-2.9] | 2.7 [2.1-3.3] | 1.7 [1.4-2.4] | 2.8 [2.3-3.4] | 2.1 [1.7-2.7] | 2.3 [1.6-2.9] | 1.2 [0.8-1.6] |
| **FEV1 (%Pred)** | 96.1 [88.2-104.7] | 84.3 [70.8-93.1] | 93.2 [84.6-102.0] | 66.8 [50.6-79.7] | 91.5 [81.6-103.0] | 73.6 [60.9-84.1] | 80.2 [63.4-94.5] | 42.5 [30.7-56.3] |
| **FVC (L)** | 3.6 [3.0-4.3] | 3.2 [2.7-4.0] | 3.7 [3.0-4.5] | 2.9 [2.3-3.7] | 3.9 [3.3-4.7] | 3.4 [2.7-4.1] | 3.7 [2.9-4.5] | 2.7 [2.2-3.4] |
| **FVC (%Pred)** | 96.0 [88.4-104.7] | 89.2 [81.4-97.8] | 95.5 [89.6-105.9] | 81.7 [69.0-93.0] | 99.3 [90.8-107.8] | 89.4 [78.8-101.5] | 95.8 [84.5-106.5] | 74.9 [62.9-87.5] |
| **FEV1/FVC** | 0.8 [0.7-0.8] | 0.7 [0.7-0.8] | 0.7 [0.7-0.8] | 0.6 [0.6-0.7] | 0.7 [0.7-0.8] | 0.6 [0.6-0.7] | 0.6 [0.5-0.7] | 0.4 [0.3-0.5] |
| **PEF** | 2.6 [1.9-3.4] | 1.7 [1.0-2.5] | 1.9 [1.2-2.7] | 0.8 [0.5-1.2] | 1.7 [1.2-2.6] | 1.0 [0.6-1.5] | 1.0 [0.5-1.8] | 0.4 [0.2-0.6] |
| **FEF25-75** | 7.4 [6.2-9.1] | 6.2 [5.0-7.7] | 7.2 [5.6-8.8] | 4.8 [3.8-6.4] | 8.1 [6.1-9.7] | 6.1 [4.8-7.3] | 6.4 [4.8-8.3] | 3.5 [2.6-4.8] |
| **%Emphysema** | 0.2 [0.0-0.5] | 0.2 [0.0-0.4] | 0.9 [0.6-1.2] | 0.9 [0.6-1.3] | 2.7 [2.1-4.4] | 3.1 [2.1-6.0] | 5.5 [3.2-14.1] | 13.9 [5.4-25.5] |
| **%Gas Trapping** | 6.0 [2.8-9.1] | 6.1 [3.2-9.6] | 18.4 [16.4-21.8] | 19.5 [16.6-24.4] | 11.8 [9.3-13.5] | 12.0 [9.8-13.6] | 23.6 [19.5-30.5] | 33.1 [26.2-39.5] |
| **Pi10 (mm)** | 1.9 [1.7-2.0] | 2.6 [2.4-3.0] | 1.8 [1.7-2.0] | 2.8 [2.5-3.3] | 1.8 [1.6-2.0] | 2.6 [2.4-2.9] | 1.9 [1.7-2.1] | 2.8 [2.5-3.1] |

Table S5 – Characteristics per CT-based phenotype
Data are presented as median [Q1-Q3 interquartile range] and no. (%).
Abbreviations: BMI, body mass index; FEF25-75, mean forced expiratory flow between 25% and 75% of FVC; FEV1, forced expiratory volume in 1 second; FVC, forced vital capacity; GOLD, Global Initiative for Chronic Obstructive Lung Diseases; PEF, peak expiratory flow; Pi10, internal perimeter of 10 mm.

|  | **Emphysema** | | | **Small airways disease** | | | **Bronchial wall thickening** | | |
| --- | --- | --- | --- | --- | --- | --- | --- | --- | --- |
|  | Entire spectrum | Mild | Moderate-severe | Entire spectrum | Mild | Moderate-severe | Entire spectrum | Mild | Moderate-severe |
| **PCA** | **0.50** | **0.17** | **0.50** | **0.60** | **0.20*** | **0.48** | 0.39 | 0.14 | 0.16 |
| **AC** | 0.35 | 0.10 | 0.39 | 0.34 | 0.16 | 0.25 | 0.10 | 0.15 | 0.06 |
| **AreaFE** | 0.41 | 0.11 | 0.41 | 0.53 | 0.15 | 0.42 | **0.45** | **0.17** | **0.19** |
| **Obstructive index** | 0.30 | 0.10 | 0.25 | 0.33 | 0.14 | 0.21 | 0.12 | 0.13 | 0.07 |
| **Peak index** | 0.40 | 0.10 | 0.32 | 0.47 | 0.14 | 0.29 | 0.24 | 0.12 | 0.08 |
| **PFT** | 0.67* | 0.27* | 0.59* | 0.65* | **0.20*** | 0.47 | 0.48* | 0.19* | 0.22* |

Table S6 – Linear regression adjusted R^2^s for different parameters derived from maximal expiratory flow-volume curves for emphysema, small airways disease and bronchial wall thickening (PRM^emph^, PRM^SAD^, Pi10 on CT) per subgroup. Results in bold indicate the best result per column between the different parameters without PFT. Asterisk is used when PFT is added to the comparison.
Abbreviations: AC, angle of collapse; AreaFE, area under the forced expiratory flow-volume loop; CT, computed tomography; PCA, principal component analysis; PFT, pulmonary function test; Pi10, airway wall thickness at an internal perimeter of 10 mm; PRM, parametric response mapping; SAD, small airways disease.

|  | **FEV1** | ***P*-value** | **FVC** | ***P*-value** | **FEV/FVC** | ***P*-value** | **FEF25-75** | ***P*-value** | **PEF** | ***P*-value** |
| --- | --- | --- | --- | --- | --- | --- | --- | --- | --- | --- |
| **PC1** | 0.76 | <0.001 | 0.49 | <0.001 | 0.74 | <0.001 | 0.82 | <0.001 | 0.71 | <0.001 |
| **PC2** | 0.14 | <0.001 | -0.11 | <0.001 | 0.49 | <0.001 | 0.17 | <0.001 | 0.34 | <0.001 |
| **PC3** | -0.02 | 0.05 | 0.07 | <0.001 | -0.15 | <0.001 | -0.21 | <0.001 | 0.20 | <0.001 |
| **PC4** | 0.04 | <0.001 | 0.07 | <0.001 | 0.01 | 0.47 | -0.19 | <0.001 | 0.14 | <0.001 |

Table S7 – Pearson correlation coefficients between the first four principal components and the classical pulmonary function parameters FEV1, FVC, FEV1/FVC, FEF25-75, PEF
Abbreviations: FEF25-75, mean forced expiratory flow between 25% and 75% of FVC; FEV1, forced expiratory volume in 1 second; FVC, forced vital capacity; PC, principal component; PEF, peak expiratory flow.


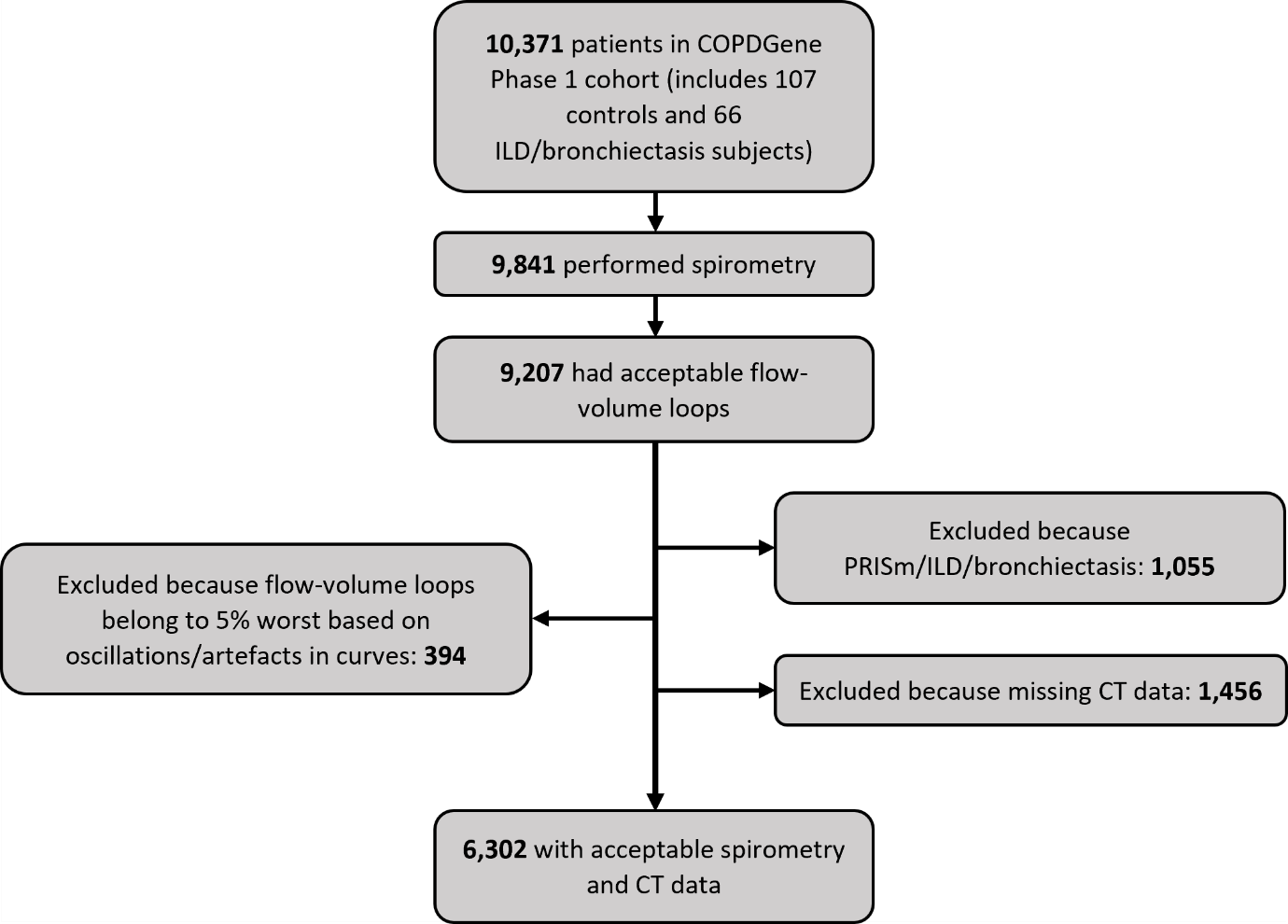


e-Figure 1 – Flow of eligible subjects for this study
Abbreviations: CT, computed tomography; COPDGene, Genetic Epidemiology of COPD; PRISm, preserved ratio impaired spirometry.
